# Associations between the brain glymphatic system and mitochondrial (dys)function: a systematic review

**DOI:** 10.3389/fnins.2025.1726054

**Published:** 2026-01-13

**Authors:** Tilda Pihala, Vesa Kiviniemi, Mika H. Martikainen

**Affiliations:** 1 Research Unit of Clinical Medicine, Neurology, University of Oulu, Oulu, Finland; 2 Oulu Functional Neuroimaging (OFNI), Research Unit of Health Sciences and Technology, University of Oulu, Oulu, Finland; 3 Department of Diagnostics Radiology, Medical Research Center, Oulu University Hospital, Oulu, Finland; 4 Biocenter Oulu, University of Oulu, Oulu, Finland; 5 Neurocenter and Medical Research Center, Oulu University Hospital, Oulu, Finland; 6 Clinical Neurosciences, Department of Clinical Medicine, University of Turku, Turku, Finland; 7 Neurocenter, Turku University Hospital, Turku, Finland

**Keywords:** glymphatic system, idiopathic intracranial hypertension, idiopathic normal pressure hydrocephalus, melatonin, mitochondria, neurodegeneration

## Abstract

**Introduction:**

Previous studies have shown that the proper functioning of both mitochondria and the glymphatic system transporting metabolites are essential for brain health. The aim of this systematic review was to identify the current evidence-based data regarding the relationship between mitochondria and the glymphatic system.

**Methods:**

This systematic review was conducted following PRISMA guidelines. The databases of PubMed, Scopus, Medline, and Web of Science were searched on June 11, 2024, for eligible studies published until June 1, 2024.

**Results:**

Of 103 studies, six were included: two original studies and four review articles. All the included studies consistently indicated that the mitochondria and the glymphatic system are likely interconnected, with evidence suggesting several potential links between them. According to original studies, mitochondrial abnormalities in idiopathic normal pressure hydrocephalus (iNPH) and idiopathic intracranial hypertension (IIH) may disrupt glymphatic system function. The included reviews highlighted REM sleep deprivation, melatonin, and inflammation as potential factors linking mitochondria and the glymphatic system.

**Discussion:**

The relationship between mitochondria and the glymphatic system is complex. Further research is needed to clarify the precise mechanisms of interaction as the current literature is largely speculative. Existing evidence suggests that mitochondrial abnormalities are present in iNPH and IIH, conditions related to impaired CSF flow and impaired glymphatic function. In addition, sleep and melatonin potentially link mitochondrial activity and the glymphatic system function and thus offer potential avenues to ameliorate disorders associated with glymphatic dysfunction by enhancing mitochondrial activity.

## 1 Introduction

Only two original human studies and four narrative reviews fulfilled our criteria; therefore, the current evidence linking mitochondrial dysfunction and glymphatic impairment is preliminary and largely hypothesis-generating. The glymphatic system is a fluid transport system that maintains cortical brain homeostasis by allowing cerebrospinal fluid (CSF) flow to remove soluble substances from the interstitial fluid into the brain parenchyma (Benveniste et al., 2020). The role of the glymphatic system in the central nervous system (CNS) is comparable to the function of the peripheral lymphatic system (Hablitz and Nedergaard, 2021) and the glymphatic system is in fact closely connected to the peripheral lymphatic system. In addition to removing metabolites, the glymphatic system probably has also other functions, such as the transport of inflowing metabolites, signalling molecules, electrolytes and waste (Bohr et al., 2022).

The glymphatic system was described in 2012 first in by Iliff & Nedergaard in mouse (Aspelund et al., 2015; Iliff et al., 2012; Louveau et al., 2015) and subsequently in the human brain (Eide and Ringstad, 2015; Kiviniemi et al., 2016). The glymphatic function is mediated by CSF flow along perivascular spaces (PVS) with the key feature of the interchange of CSF with interstitial fluid (ISF) between astrocyte end-feet gaps over the blood brain barrier glia limitans (Benveniste et al., 2020). The aquaporin-4 (AQP4) channels mediate in the astrocytic end-feet size that is assumed to function as a mechanistic valve governing the transition from awake state to increased hydrodynamic flushing during sleep (Yankova et al., 2021).

CSF is primarily produced by ependymal cells in the choroid plexuses of the cerebral ventricles, and it is responsible for the glymphatic circulation (Wang et al., 2023). The total CSF volume of a human adult is about 150 mL, which is circulated 3–4 × per day. CSF flow in the brain consists of three components: the para-arterial inflow pathway, the mixing with ISF within the brain parenchyma, and the paravenous CSF-ISF outflow pathway surrounding the veins (Rasmussen et al., 2022). The CSF-ISF mixture from the paravenous pathway enters the venous circulation either indirectly through the lymphatic system in the paraspinal/neural routes, or via transdural routes directly into the superior sagittal sinus (Hablitz and Nedergaard, 2021). Ultimately, metabolic waste products of the brain are transferred to the liver and kidneys (Nedergaard, 2013). Also, periarterial clearance driven by vasomotion has been proposed (Carare et al., 2020).

Diffusion and advection are transport mechanisms responsible for the removal of soluble metabolic waste from brain parenchyma depending on the perivascular space location (Thomas, 2019). Advection is a directed process in which solutes are transported by the flow of a liquid within a confined space such as the perivascular space. In contrast, diffusion is a less directed movement of molecules driven by the random Brownian motion of water molecules in a freer environment. Dispersion of the combination of these two occuring at the intersections of convective flow PVS’s and intraparenchymal ISF spaces. Astrocytes and blood vessels influence the shape of the PVS, thus controlling the movement of perivascular fluid (Hablitz and Nedergaard, 2021; Bojarskaite et al., 2023; Hauglund et al., 2025). Glymphatic system function depends additionally on AQP4 astroglial water channels normally situated at the vessel wall glia (Bohr et al., 2022). Perivascular AQP4 channels enable CSF influx into the astrocytes governing their volume which controls the inter-astrocytic clef that then mediates the in/outflow between CSF-ISF. Moreover, the state of arousal affects the glymphatic flow: glymphatic solute transport is increased during sleep compared to wakefulness by norepinephrine induced infraslow hydrodynamic brain oscillations driven by vasomotor waves (Nedergaard, 2013; Hauglund et al., 2025; Xie et al., 2013; Elabasy et al., 2025).

Physiological pulsations drive the glymphatic advection and convection. Each pulsation is always produced by muscle action; arterial smooth muscle vasomotion drives Mayer 0.03 Hz waves and slow control gain of heart-lung flow control causes Traube-Hering 0.1 Hz waves. Respiratory 0.3 Hz brain pulsations that are produced by diaphragm & intercostal respiratory muscles and carried out into the intracranial space via incompressible fluids like venous blood and spinal canal CSF; finally, the heart induces arterial 1 Hz pulsatility that also induces the perfusion pressure that drives the blood flow. All these together drive central nervous system CSF flow (Kiviniemi et al., 2016; Raitamaa et al., 2021).

The brain also has a high metabolic rate, which leads to significant production of metabolites (Kopeć et al., 2023). The pulsatile CSF flow rebalances fluid level, potassium, and metabolic waste products, such as lactate and amyloid beta, from the interstitial space (Hablitz and Nedergaard, 2021). Effective removal of neurotoxic substances, such as amyloid-beta and tau protein, is essential for brain health. Disruption of the glymphatic system leads to these proteins, associated with progressive loss of neurons characteristic of neurodegenerative disorders such as Alzheimer’s disease (AD) (Nedergaard and Goldman, 2020).

Mitochondria are ubiquitous organelles found in eukaryotic cells and play a crucial role in the production of adenosine triphosphate (ATP) for the cellular aerobic energy needs. Mitochondria are also central in maintaining cellular homeostasis (Harrington et al., 2023; Suomalainen and Nunnari, 2024). In addition to energy metabolism, mitochondria are involved in several other key processes including calcium signalling and homeostasis, apoptosis, cell signalling, and the production of reactive oxygen species (ROS). ROS are produced particularly by complexes I and III of the electron transport chain as by-products of the cellular respiration (Gorman et al., 2016). Excessive production of ROS can lead to oxidative stress, which is harmful to neurons. There is considerable variability in the morphology, function, and intracellular location of mitochondria (Pekkurnaz and Wang, 2022). Neurons are particularly dependent on the diversity of mitochondria and their distribution between dendrites and axons. Pathological changes in mitochondrial function and morphology are associated with the development and progression of several neurodegenerative diseases, which are among the most significant challenges in the field of neurology (Kopeć et al., 2023).

Mitochondrial diseases are a clinically heterogeneous group of genetic disorders, with clinical manifestations varying in symptoms, onset, and severity (Gorman et al., 2016). The common underlying cause of mitochondrial diseases is the disruption of mitochondrial oxidative phosphorylation. Respiratory chain dysfunction, caused by mutations in either mitochondrial DNA (mtDNA) or nuclear DNA, leads to defective ATP production, energy deficiency, and disrupted cellular function (Suomalainen and Nunnari, 2024; Gorman et al., 2016). This is why symptoms of mitochondrial diseases typically appear in tissues with high energy demands, such as the brain.

As all cellular processes depend on mitochondrial ATP production, it is conceivable that the function of the glymphatic system would also be affected by mitochondrial dysfunction. All pulsations driving the CSF are produced by muscle work that is mediated within vessels and spinal canal and the cranium. However, the details of how mitochondria are involved in the glymphatic system, and how it might be affected in mitochondrial disorders are unexplored. Interestingly, a recent study revealed hypoxic pockets in the mouse cortex interstitium, that may prove a mechanistic link between lack of physical exercise, sedentary lifestyle, and the risk of dementia (Beinlich et al., 2024). It is intriguing to hypothesize that variation in cortical mitochondria may be closely related to these processes. To create a basis for future research on this topic, we decided to do a systematic review to explore what is known about the interaction between mitochondria and the glymphatic system.

## 2 Methods

This systematic review follows the guidelines outlined in the Preferred Reporting Items for Systematic Reviews and Meta-Analyses (PRISMA) statement (Page et al., 2021). A comprehensive online literature search was conducted using Scopus, PubMed, Medline, and Web of Science databases on 11 June 2024.

The database search included the following items: “glymphatic system,” “glymphatic pathway,” “glymphatic clearance system,” “meningeal lymphatic vessel*,” “brain perivascular space*,” “Virchow-Robin space*,” “mitochondri*,” “oxidative phosphorylation,” “OXPHOS,” and “electron transport chain deficienc*.” The PubMed search also included the following MeSH (Medical Subject Headings) terms “glymphatic system,” “mitochondria,” “mitochondrial diseases,” and “oxidative phosphorylation.”

Studies were included if they met the following criteria: (1) reporting original research, (2) published before June 1, 2024, (3) published in English, (4) indexed in PubMed, and (5) available abstract. After the initial search, the inclusion criterion (1) was expanded to include review articles due to the small number of identified studies reporting original data.

The first author (TP) performed the database searches and the screening of the titles and abstracts. In uncertain cases, full texts were scrutinised to decide on inclusion or exclusion. All uncertain decisions to include or exclude papers were double-checked with the last author (MM) to reach consensus through discussion.

## 3 Results

The methodological quality of all included studies was assessed independently by two reviewers. Discrepancies were resolved through discussion. The most common concerns were related to small sample sizes, observational design, and the narrative nature of reviews. Despite these limitations, the included evidence was considered adequate in quality for addressing the review question in a hypothesis-generating way, as well as in underlining the need for further study. Moreover, we note that all the included reviews were narrative rather than systematic. Findings should therefore be interpreted with reasonable caution.

**Figure 1:**
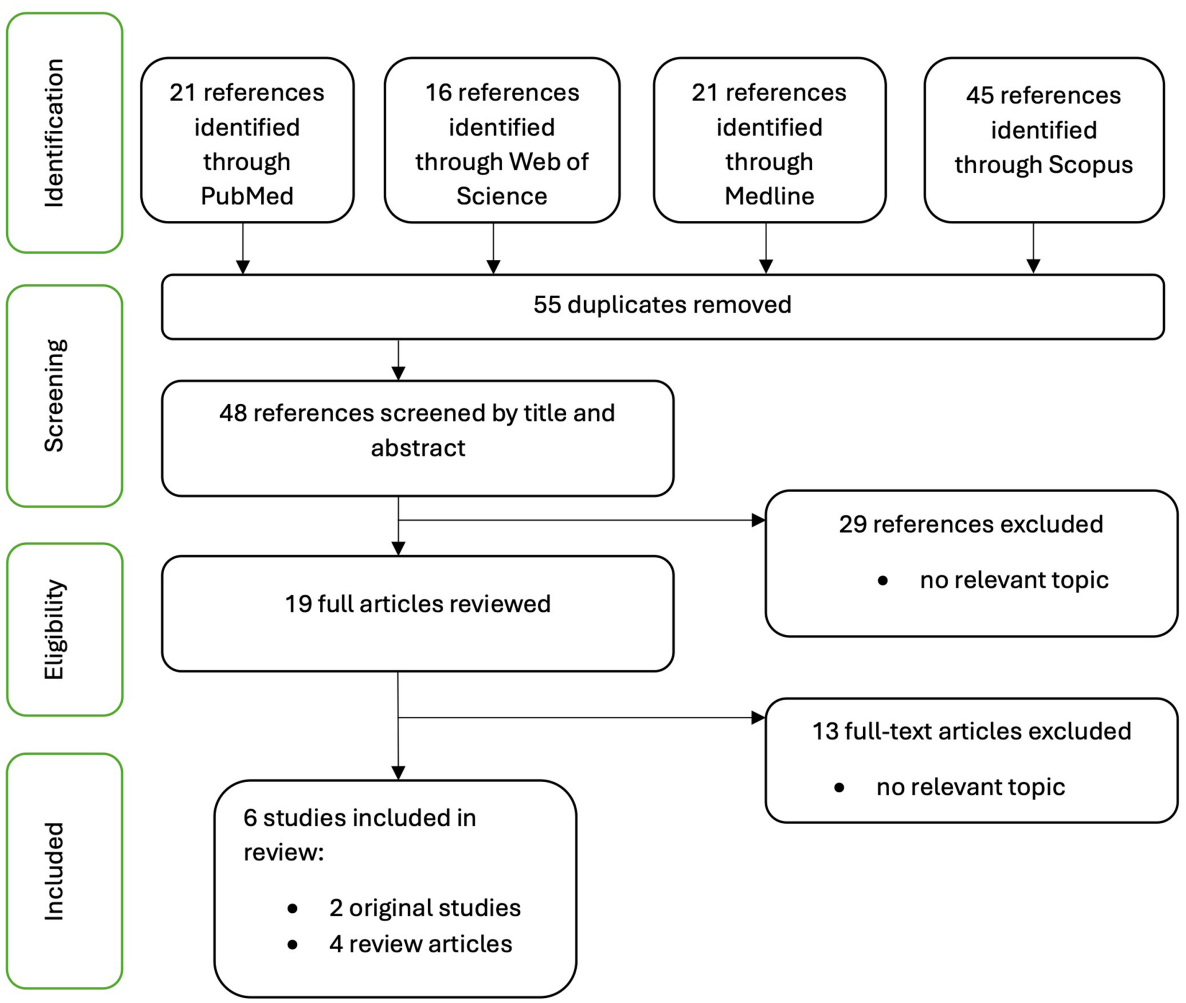
Flowchart of the literature search.

The database search (Figure 1) yielded *N* = 103 articles, of which six were included in the final review. Two of the articles were original studies (Eide et al., 2021; Hasan-Olive et al., 2019), and four were review articles (Kopeć et al., 2023; Salehpour et al., 2022; Trigo et al., 2023; Nakagawa and Yamada, 2023). The key findings of the studies are presented in Table 1. After removing duplicates (*n* = 55), screening was performed based on the title and abstract (*n* = 48), followed by full-text screening (*n* = 19).

**Table 1:** The identified studies on the associations of mitochondrial (dys)function and the glymphatic system.

Study	Study type	Population/sample	Main mitochondrial measure	Main glymphatic/CSF measure	Relevance to mitochondria–glymphatic link
Eide et al. (2021)^*^	Original study	Cortical brain biopsies from adults with IIH (*n* = 8) and reference individuals without IIH (*n* = 9).	Mitochondrial structural abnormalities: swollen appearance, cristae disruption, and reduced electron density on TEM.	Degree of astrogliosis evaluated by GFAP immunoreactivity.	Patients with IIH had increased pathological mitochondria in the perivascular astrocyte end-feet compared to reference patients. The degree of astrogliosis correlated with the number of defective mitochondria. Through impairing astrocyte regulation and signalling, dysfunctional mitochondria may impair glymphatic system function.
Hasan-Olive et al. (2019)^*^	Original study	Cortical brain biopsies from adults with iNPH (*n* = 30) and reference individuals without iNPH (*n* = 9).	Mitochondrial structural abnormalities: swollen appearance, cristae disruption, and reduced electron density on TEM.	Perivascular AQP4 expression and degree of astrogliosis, assessed by light microscopy immunohistochemistry.	Patients with iNHP had more pathological mitochondria in the perivascular astrocyte end-foot processes compared to controls. The number of pathological mitochondria correlates negatively with perivascular AQP4 expression, which is essential for glymphatic clearance.
Salehpour et al. (2022) (review)^*^	Review	Animal studies on the neurotherapeutic benefits of PBM therapy on glymphatic drainage and clearance.	No single primary mitochondrial outcome: Describes PBM-induced mitochondrial responses, e.g., mitochondrial membrane potential.	No single primary glymphatic/CSF outcome: Narrative synthesis of glymphatic drainage indicators.	PBM treatment increases brain oxygen saturation and enhances mitochondrial ATP production, and thus may promote glymphatic outflow.
Trigo et al. (2023) (review)^*^	Review	Animal and human studies on neurodegenerative disorders.	No single primary mitochondrial outcome: Narrative synthesis of multiple mitochondrial parameters, e.g., mitophagy, oxidative stress, bioenergetic function.	No single primary glymphatic/CSF outcome.	Mitochondrial dysfunction can impair glymphatic clearance, which in turn may contribute to neurodegeneration.
Nakagawa and Yamada (2023)^*^	Review	Animal and human studies on psychiatric and neurodegenerative disorders focusing on metal homeostasis, mitochondria, and the locus coeruleus.	No single primary mitochondrial outcome: Narrative synthesis of mitochondrial dysfunction related to metal dyshomeostasis.	No single primary glymphatic/CSF outcome.	Metal dyshomeostasis in the CSF may result in mitochondrial dysfunction in the glial cells of the locus coeruleus, which may further impair the glymphatic system function.
Kopeć et al. (2023)^*^	Review	Animal and human studies on neurodegenerative disorders, focusing on glymphatic system and mitochondrial dysfunction.	No single primary mitochondrial outcome: Narrative synthesis of multiple mitochondrial parameters, e.g., ATP production, ROS generation.	No single primary glymphatic/CSF outcome: Narrative synthesis of glymphatic dysfunction indicators, e.g., AQP4 expression and polarization.	REM sleep deprivation is linked to both disrupted mitochondrial bioenergetics and the glymphatic system dysfunction. Melatonin provides protective effects on astrocyte mitochondria, while mitochondrial ROS production may disrupt astrocyte function and subsequently impair glymphatic clearance. Mitochondrial energy production is crucial for CSF production and the proper functioning of the glymphatic system.

AQP4, aquaporine 4; ATP, adenosine triphosphate; CSF, cerebrospinal fluid; GFAP, glial fibrillary acidic protein; IIH, idiopathic intracranial hypertension; iNPH, idiopathic normal pressure hydrocephalus; PBM, photobiomodulation; ROS, reactive oxygen species; TEM, transmission electron microscopy.

*Reference number in the manuscript reference list.

The two original studies were prospective observational studies. The other (Eide et al., 2021) investigated mitochondrial changes in astrocytic end-feet in brain biopsies of patients with idiopathic intracranial hypertension (IIH), while the other (Hasan-Olive et al., 2019) examined altered mitochondrial phenotypes in neurons and astrocytes in patients with idiopathic normal pressure hydrocephalus (iNPH). In both studies, transmission electron microscopy was used. The review articles focused on various aspects of mitochondrial dysfunction, glymphatic system impairment, and their relationship to neurodegeneration. Salehpour et al. (2022) synthesized preclinical and experimental animal studies on photobiomodulation therapy, highlighting its potential to enhance brain lymphatic drainage and mitochondrial function. A review by Trigo et al. (2023) explored the role of mitochondrial dysfunction in neurodegenerative diseases. Nakagawa and Yamada (2023) explored the influence of metal dyshomeostasis on mitochondrial dysfunction in the locus coeruleus and its subsequent effects on the glymphatic system. Kopeć et al. (2023) reviewed how the glymphatic system and mitochondrial dysfunction contribute to neurodegeneration and how they may interact to exacerbate disease progression.

Previous research suggests that the glymphatic system is impaired in iNPH (Eide and Ringstad, 2019; Ringstad et al., 2017). When examining neurons and the end-foot processes of perivascular astrocytes using transmission electron microscopy, pathological mitochondria were observed in a greater number in iNPH patients compared to reference patients (Hasan-Olive et al., 2019). In addition, iNPH patients had a reduced number of normal mitochondria in the end-feet of astrocytes compared to reference patients. There was also evidence of a positive correlation between the number of normal mitochondria and perivascular AQP4 expression. In contrast, the number of pathological mitochondria was negatively correlated with perivascular AQP4 expression. The reduced number of normal mitochondria and the increased number of pathological mitochondria may impair astrocytic energy homeostasis, which could then negatively affect the glymphatic system.

Glymphatic system dysfunction is also implicated in IIH (Bouffard et al., 2024; Schartz et al., 2024). Electron microscopy revealed an increased number of pathological mitochondria and a reduced number of normal mitochondria in the end-feet of perivascular astrocytes also in patients with IIH (Eide et al., 2021). Additionally, the degree of astrogliosis was found to correlate positively with the number of pathological mitochondria and negatively with the number of normal mitochondria in the astrocytic perivascular end-foot processes. These observations suggest that pathological mitochondria may impact the regulation and signalling of astrocytic end-feet and thus contribute to the glymphatic system dysfunction in IIH patients.

One of the identified review articles focused on the effects of photobiomodulation (PBM) therapy on the meningeal lymphatic vessels (MLVs), that are considered a component of the glymphatic system (Salehpour et al., 2022). PBM is a non-invasive therapy in which cellular functions can be stimulated using visible and near-infrared light. The review described how beneficial effects of PBM on mitochondria could affect the glymphatic system: PBM increases oxygen saturation in the brain, leading to enhanced mitochondrial ATP production. The increased ATP availability might then further improve the contractility of MLVs, thereby increasing the outflow of the glymphatic system.

A recent review analysed the role of mitochondria in neurodegeneration and its treatment (Trigo et al., 2023). According to this paper, research suggests that metabolic activity affects the functioning of the glymphatic system and that there might be connections between mitochondrial function and the glymphatic system.

Another recent review suggested some pathogenic mechanisms that may result in neurodegeneration (Nakagawa and Yamada, 2023). Metal dyshomeostasis in the cerebrospinal fluid may result in mitochondrial dysfunction in the glial cells of the locus coeruleus. This may further lead to locus coeruleus hyperactivity, resulting in disturbances in the sleep–wake cycle and REM sleep, and the impairment of the glymphatic system function.

**Figure 2:**
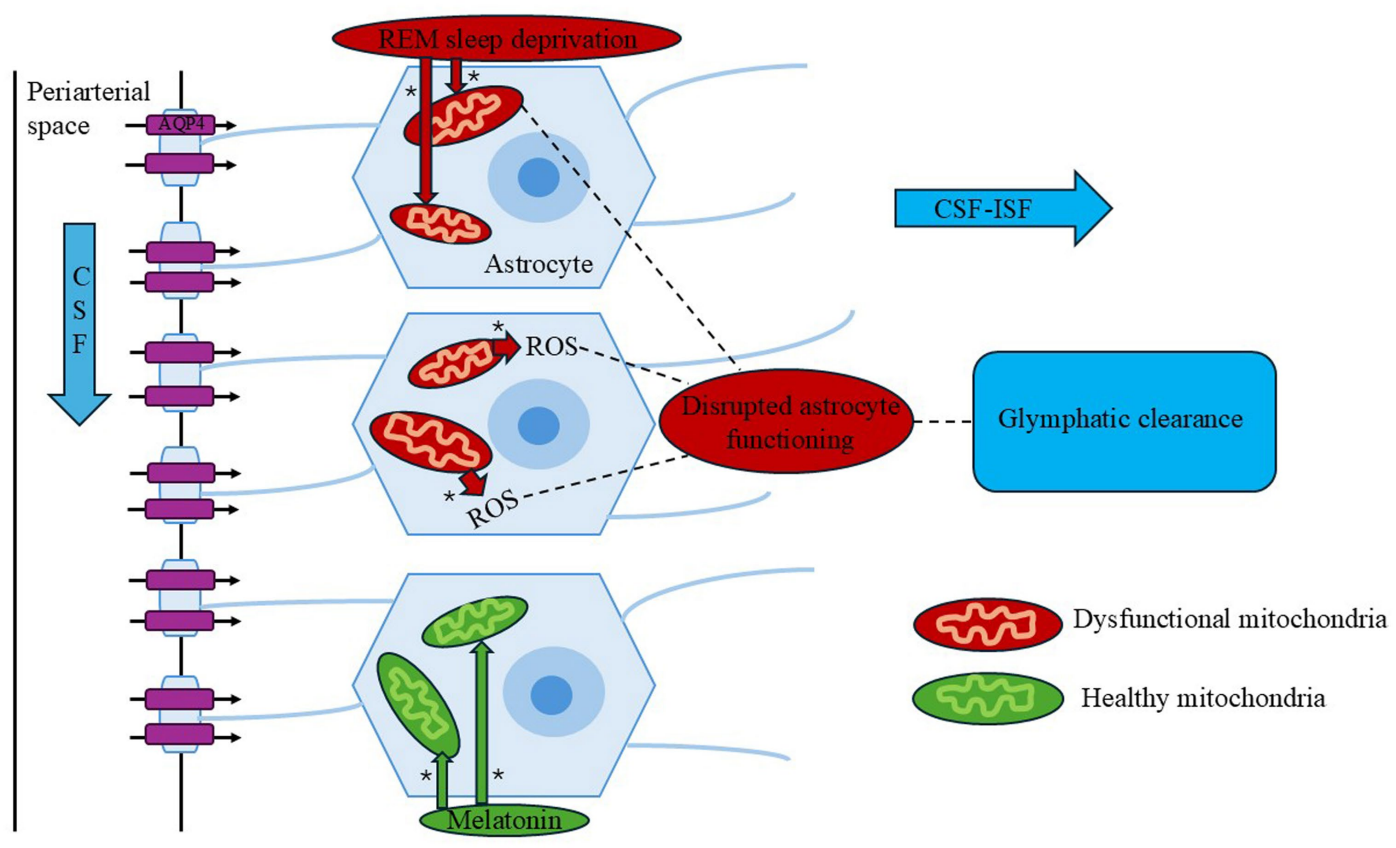
Illustration of links between mitochondrial (dys)function and the glymphatic system at the astrocyte level. This figure represents a conceptual hypothesis model synthesizing the findings of the included reviews. It does not represent any single experimentally proven pathway. Melatonin protects mitochondria, whereas REM sleep deprivation impairs astrocytic mitochondrial function. Increased ROS production by impaired mitochondria results in neuroinflammation that can further disrupt astrocyte function and impair glymphatic clearance. AQP4, aquaporin 4; CSF, cerebrospinal fluid; ISF, interstitial fluid; REM, rapid eye movement; ROS, reactive oxygen species. *Supported by animal or mechanistic evidence.

Kopeć et al. (2023) focused on the relationship between the glymphatic system and mitochondria in the context of neurodegenerative disorders. This review suggested dysfunction in both mitochondria and the glymphatic system leads to accumulation of protein aggregates that are characteristic of neurodegeneration. These cascades are probably not independent; thus, there may be a crucial interaction between mitochondrial and glymphatic system dysfunction in the pathogenesis of neurodegenerative diseases. In addition to protein aggregates, REM sleep deprivation is a factor that links impaired mitochondrial bioenergetics and glymphatic system dysfunction, as it can cause both. The links between mitochondrial (dys)function and the glymphatic system at the astrocyte level are illustrated in Figure 2. This review also underlined the significance of mitochondrial energy production for the glymphatic system. Disruptions in mitochondrial energy metabolism can affect CSF production, as the osmotic gradient required for this process relies on ATP derived from mitochondrial OXPHOS or glycolysis. Furthermore, melatonin plays a role in the interaction between mitochondria and the glymphatic system. Its protective effects target the mitochondria of astrocytes, suggesting a potential positive impact on glymphatic clearance. In addition to protein aggregates, REM sleep deprivation, mitochondrial energy production, and melatonin, Kopeć et al. (2023) explored inflammation as another factor connecting mitochondria and the glymphatic system. ROS produced as mitochondrial by-products may trigger NLRP3 inflammasome activation, linked to microglial activation. Microglial activation could then disrupt astrocyte functioning, leading to dysfunction in the glymphatic system.

## 4 Discussion

Overall, there are currently few published studies on this topic, and most of those that were included in this study addressed the connection between mitochondria and the glymphatic system speculatively. Further research is warranted to gain a more detailed and evidence-based understanding of the connections between mitochondria and the glymphatic system.

The identified original studies suggest that mitochondrial abnormalities in astrocytic end-feet may disrupt the glymphatic system, potentially impairing the clearance of waste products and contributing to the pathophysiology observed in both iNPH and IIH (Eide et al., 2021; Hasan-Olive et al., 2019). To support this suggestion, it would be beneficial to investigate the impact of mitochondrial dysfunction on perivascular astrocytes and, consequently, on the functioning of the glymphatic system, as these are currently not well understood. The reviews included in this study highlight several potential links between mitochondrial function and the glymphatic system. These include ATP production (Kopeć et al., 2023; Salehpour et al., 2022; Trigo et al., 2023), sleep (Kopeć et al., 2023; Nakagawa and Yamada, 2023), melatonin, protein aggregation, and inflammation (Kopeć et al., 2023).

Although the exact mechanisms are yet to be clarified, it seems reasonable to suggest a connection between mitochondrial function, particularly aerobic energy production, and the physiological functioning of the glymphatic system. As the dysfunction of the glymphatic system is increasingly implicated in several devastating neurological disorders, the identification of potential ways to maintain and improve the glymphatic function would be of importance. Maintaining and enhancing mitochondrial function in the brain could be one such avenue. Moreover, recent findings suggest that cortical hypoxic pockets may prove a mechanistic link between lack of physical exercise, sedentary lifestyle, and the risk of dementia (Beinlich et al., 2024). Cortical mitochondria may be closely related to these processes.

Although no studies have yet tested effects on glymphatic metrics directly, hypothetical routes through which melatonin, NAD+/nicotinamide riboside, and exercise might influence the glymphatic system can be proposed based on their known effects on mitochondria and sleep. Melatonin has positive effects on mitochondrial function (Cardinali and Vigo, 2017). It can protect mitochondria from amyloid-beta toxicity as well as from oxidative damage caused by free radicals. In addition to its own free radical scavenging activity, melatonin activates other antioxidants (Melhuish Beaupre et al., 2021). Melatonin levels are reduced with aging, and this reduction is associated with mitochondrial dysfunction in the brain (Petrosillo et al., 2008). Melatonin supplementation has been suggested to have beneficial effects in neurodegenerative diseases such as Alzheimer’s disease (Steinbach and Denburg, 2024). Melatonin supplementation has also been suggested to assist in the maintenance of normal mitochondrial function with ageing (Reiter et al., 2024). The central role of melatonin in the regulation of circadian rhythm is well established (Sack et al., 2000). Interest in the potential therapeutic role of melatonin is increasing as disordered sleep has emerged as an important early symptom associated with neurodegenerative diseases such as Alzheimer’s disease (Khandayataray and Murthy, 2025; Parhizkar and Holtzman, 2025; Videnovic and Cai, 2025). It remains to be determined whether melatonin deficiency could impair the functioning of the glymphatic system through its impact on mitochondria. Conversely, it is not yet established whether melatonin supplementation could be useful in enhancing brain mitochondrial function and the glymphatic system.

In addition to melatonin-related pathways, mitochondria are also involved in other mechanisms that influence sleep (Davinelli et al., 2024). For example, there is a bidirectional relationship between redox signalling and sleep. Disrupted sleep has been linked to increased ROS production and oxidative stress can negatively impact circadian rhythm regulation through modifications in adenosine monophosphate kinase (AMPK) signalling. Additionally, sleep deprivation may impair mitochondrial function and lead to reduced ATP production, further affecting cellular energy balance and physiological homeostasis. Sleep loss is increasingly perceived as a metabolic disorder that is associated with neurodegenerative disease such as Alzheimer’s disease and Parkinson’s disease (Feeney et al., 2025). Key characteristic of both these conditions is a reduction in energy production caused by mitochondrial dysfunction (Song et al., 2022). Because sleep disturbances have been shown to impair glymphatic function, investigating how mitochondrial properties and activity influence sleep physiology may offer important insights into the role of mitochondria in regulating glymphatic activity (Kopeć et al., 2023). Literature on sleep problems in patients with IIH is quite limited. Based on previous studies, sleep disturbances are common among adolescents with IIH (Tokatly Latzer et al., 2023). In addition, obstructive sleep apnoea seems more prevalent in patients with IIH than in healthy controls (Kentis et al., 2025). Previous studies also suggest that iNPH is associated with sleep disturbances, particularly obstructive sleep apnoea, associated with impaired glymphatic function (Román et al., 2019).

Nicotinamide adenine dinucleotide (NAD+) regulates mitochondrial function (Zhan et al., 2023). As a precursor of NAD+, nicotinamide riboside (NR) supplementation may help enhance mitochondrial health, for example, by influencing mitochondrial autophagy and division (Wang et al., 2024). This system is increasingly appreciated as a potential pathway to treat several neurodegenerative brain disorders (Lautrup et al., 2019) and several studies support the beneficial effect of nicotinamide supplementation on mitochondrial function, particularly in muscle (Elhassan et al., 2019; Zhang et al., 2016). Mitochondrial dysfunction is also observed in obesity: brain insulin resistance causes oxidative stress and reduces ATP production (Cai et al., 2024). In contrast, physical exercise has positive effects on insulin sensitivity and mitochondrial health by reducing oxidative stress, maintaining proper mitochondrial structure, and enhancing energy metabolism (Sun et al., 2022; Vargas-Mendoza et al., 2021). Therefore, weight management and physical activity might be potential methods for improving even glymphatic system function.

Based on this systematic review, we conclude that the potential connections between mitochondrial (dys)function and the brain glymphatic system remain largely unexplored. Existing evidence suggests that mitochondrial abnormalities are present in iNPH and IIH, conditions related to impaired CSF flow and impaired glymphatic function. In addition, sleep and melatonin potentially link mitochondrial activity and the glymphatic system function and thus offer potential avenues to ameliorate disorders associated with glymphatic dysfunction by enhancing mitochondrial activity. Better understanding of these mechanisms might help us develop new therapeutic strategies for several conditions that are presently lacking in effective treatments.

Several important questions remain to be explored regarding the relationship between mitochondria and the glymphatic system. Key areas of future research include more detailed understanding of how mitochondrial defects in astrocytes influence glymphatic influx, interstitial fluid clearance, and efflux *in vivo*. Specifically, it will be essential to determine how ROS generated by dysfunctional mitochondria affect the localization and function of AQP4 channels. Further study is also needed to clarify whether interventions such as melatonin or NR supplementation, exercise, or other types of mitochondrial metabolic modulation could improve glymphatic system function and so provide therapeutic benefits.

This study has some limitations. There is only human histological data available. There are no interventional or longitudinal studies demonstrating a link between targeted mitochondrial modulation and glymphatic readouts. Additionally, most mechanistic links are inferred from separate literatures. Language and database restrictions may have led to omission of some relevant reports.

## Data Availability

The datasets presented in this article are not readily available because no new data were generated; all data are extracted from previously published sources cited in the reference list. Requests to access the datasets should be directed to MM, mika.martikainen@oulu.fi.
